# The Galveston Sacrifice

**Published:** 1900-12

**Authors:** 


### THE GALVESTON SACRIFICE.

It may seem late to appeal to the dentists of this country for financial aid to our professional brethren sufferers in that fearful inundation now so sadly familiar to all, but it was supposed that professional men were aiding, with their fellow-citizens in other walks of life, to swell the funds being raised, and that to the extent of their financial ability. This was the fact within the writer's immediate observation, as it was presumably so elsewhere.

It is now officially stated that the dentists of that city will not be able to participate in the general fund, as that has been devoted to feeding and clothing and removal of wreckage, and, in any event, is not sufficient to cover more than five per cent. of the loss.

Subscriptions can be sent direct to Dr. D. S. Killough, secretary, 2123 Market Street, Galveston, Tex.

